# Sphingosine 1-phosphate attenuates MMP2 and MMP9 in human anaplastic thyroid cancer C643 cells: Importance of S1P_2_

**DOI:** 10.1371/journal.pone.0196992

**Published:** 2018-05-07

**Authors:** Muhammad Yasir Asghar, Kati Kemppainen, Taru Lassila, Kid Törnquist

**Affiliations:** 1 Cell Biology, Faculty of Science and Engineering, Åbo Akademi University, BioCity, Artillerigatan, Turku, Finland; 2 The Minerva Foundation Institute for Medical Research, Biomedicum Helsinki, Helsinki, Finland; University of South Alabama Mitchell Cancer Institute, UNITED STATES

## Abstract

In anaplastic thyroid cancer C643 cells, sphingosine 1-phosphate (S1P) attenuates migration by activating the S1P_2_ receptor and the Rho-ROCK pathway. In the present study, we show that stimulating C643 cells with S1P decreases the expression, secretion and activity of matrix metalloproteinase-2 (MMP2), and to a lesser extent MMP9. Using receptor-specific antagonists, and S1P_2_ siRNA, we showed that the inhibition of expression of MMP2 is mediated through S1P_2_. Furthermore, S1P inhibited calpain activity, and inhibiting calpain pharmacologically, inhibited the effect of S1P on MMP2 expression and activity, and on MMP9 activity. S1P treatment increased Rho activity, and by incubating cells with the Rho inhibitor C3 transferase or the ROCK inhibitor Y27632, the S1P-induced inhibition of invasion and MMP2 expression and activity was abolished. We conclude that S1P attenuates the invasion of C643 cells by activating S1P_2_ and the Rho-ROCK pathway, by decreasing calpain activity, and by decreasing the expression, secretion and activity of MMP2 and, to a lesser extent, MMP9. Our results thus unveil a novel function for the S1P_2_ receptor in attenuating thyroid cancer cell invasion.

## Introduction

Sphingosine 1-phosphate (S1P), a bioactive lipid, is a regulator of many cellular processes, including cancer cell invasion and migration [1]. S1P can bind to five G-protein coupled receptors (S1P_1-5_), which activate downstream signaling pathways [2]. In many cancer cells, including follicular thyroid cancer ML-1 cells, S1P promotes migration and invasion by activating S1P_1,3_ and downstream PI3K-Akt and Rac signaling pathways [3–8]. However, S1P inhibits migration and invasion by activating S1P receptor 2 and the downstream Rho-ROCK signaling pathway and by inhibiting Rac activity in many cell types [9], including human anaplastic thyroid cancer C643 cells [10]. In some cell types, however, S1P_2_ can also enhance migration [11]. The small GTP-ase Rac promotes invasion [12] and has been shown to regulate S1P-evoked matrix metalloproteinase 2 and -9 (MMP2 and -9) secretion in cancer cells [13]. The MMPs are zinc-dependent proteolytic enzymes, which are expressed and secreted into the extracellular matrix by cancer cells [14]. The inactive zymogen forms of MMP2 and MMP9 are activated by calpains (calcium-dependent proteolytic enzymes), which cleave the pro-peptide domains. MMP2 and MMP9 use mainly collagen IV as substrate and digest the basement membrane to promote cell invasion in cancer cells [13,15–18].

Previous studies show that increased expression and activity of MMP2 and MMP9 in thyroid cancer cells promotes invasion [18]. Recently, we have reported that S1P induces secretion and activity of MMP2 and MMP9 through S1PR_1,3_, and that these MMPs are important for the S1P-evoked invasion of thyroid cancer ML-1 cells [19]. However, the role of MMP2 and MMP9 in S1P-evoked inhibition of invasion of thyroid cancer cells remained unknown. In the present study, we have investigated the expression, secretion and activity of MMP2 and MMP9 in thyroid cancer C643 cells where S1P inhibits invasion. Our results show for the first time that S1P can attenuate the expression, secretion and activity of MMP2 and MMP9. This occurs via a S1P_2_-evoked activation of the Rho-ROCK pathway, and an inhibition of calpain activity. We propose that S1P-evoked inhibition of invasion is mediated, at least in part, by effects on MMP2 and MMP9.

## Materials and methods

DMEM, BSA, fatty acid-free BSA (FAF-BSA), Mitomycin C, ethidium bromide, SB-3CT and JumpStart Taq DNA polymerase were purchased from Sigma Aldrich Corporation (St. Louis, MO, USA). RPMI 1640 medium was from Lonza (Basel, Switzerland). S1P was purchased from Biomol (Plymouth, PA, USA). FBS, penicillin/streptomycin (P/S), L-Glutamine, trypsin, F-12 Ham’s nutrient medium and OptiMEM were from Gibco Life Technologies (Grand Island, NY, USA). Primary antibodies against S1P_2_ and S1P_3_ were purchased from Santa Cruz Biotechnology, Inc. (Santa Cruz, CA, USA). HRP-conjugated goat anti-rabbit IgG was purchased from Bio-Rad Laboratories (Hercules, CA, USA). MMP2 and anti-mouse IgG antibodies were from Cell Signaling Technology (Denver, MA, USA). MMP9, S1P_1_, S1P_4_, S1P_5_ antibodies and the calpain activity assay kit were purchased from Abcam (Cambridge, MA, USA). Human collagen type IV and cell culture plastic-ware were from Becton Dickinson (Bedford, MA, USA). Transwell migration inserts were from Corning, Inc. (Corning, NY, USA). The bicinchoninic acid protein assay reagent kit was from Pierce. All the chemicals and reagents used were of molecular biology and reagent grades. JTE-013 was from Tocris Biosciences (Ellisville, MO, USA). VPC23019 was purchased from Avanti Polar Lipids (Alabuster, AL, USA). Y27632 was obtained from Calbiochem (San Diego, CA, USA). C3 Transferase was from Cytoskeleton, Inc. (Denver, CO, USA). MMP2 siRNA “ACAAGAACCAGAUCACAUA” and MMP9 siRNA “GCAUAAGGACGACGUGAAU” were ON-TARGET SMART pool siRNA from Dharmacon (Lafayette, CO, USA). Nitrocellulose transfer membrane was from Whatman (Maidstone, UK). The western lightning Plus-ECL kit was from Perkin Elmer (Whatman, MA, USA).

### Cell culture

The human anaplastic thyroid cancer C643 cells were provided by Dr. Nils-Erik Heldin (Karolinska Institute, Stockholm, Sweden). The cells were grown in DMEM supplemented with 10% FBS, 1% P/S and 2 mM L-glutamine. The human follicular thyroid cancer FTC-133 cells were obtained from Banca Biologica e Cell Factory, National Institute of Cancer Research (Genova, Italy). The cells were cultured in DMEM and F12 (Ham’s nutrient) medium (1:1) supplemented with 10% FBS, 1% P/S and 2 mM L-glutamine. Both C643 and FTC-133 thyroid cancer cell lines used in this study are verified unique thyroid cancer cell lines [20]. The cell cultures were maintained in a water-saturated atmosphere with 5% CO_2_ at 37C°.

### End-point PCR and quantitative real-time PCR (qPCR)

RNA was extracted with TRI Reagent (Sigma Aldrich; St Loius, MO) and Aurum^TM^ Total RNA Mini Kit (Bio-Rad; CA, USA) according to the manufacturer’s instructions. RNA integrity was checked by gel electrophoresis and RNA concentration and purity was determined with Nanodrop 2000 (Thermo Fisher Scientific; Waltham, MA) and NanoVue Plus (Healthcare Bio-Sciences AB; Uppsala, SE). cDNA samples were prepared with RevertAid reverse transcriptase and SuperScript IV ® Reverse Transcriptase Invitrogen (Thermo Fisher Scientific, MA, USA) from equal amounts of RNA. For MMP9 qPCR experiments on FTC-133 RNA samples, the cDNA was made by using MMP9 gene specific primer instead of random hexamers.

End-point PCR- The reaction mixtures for S1P_1-5_ were prepared with JumpStart^TM^ Taq polymerase according to the manufacturer’s instructions (Sigma-Aldrich, MO, USA). Reaction mixtures without the reverse transcriptase enzyme RNA controls (RNA C) and Non-template controls (NTC) were used to ensure that RNA preparations and PCR mixtures were not contaminated. RT-PCR were performed with Mastercycler gradient (Eppendorff, Hamburg, Germany). Human hypoxanthine phosphoribosyl transferase (HPRT) was used as a reference gene. The information about primers sequences and the PCR cycling settings are mentioned in the Table 1.

**Table 1:** PCR primers, probes and cycling conditions.

Gene	Primer Sequence	Probe	Amplicon	MgCl2, mM	Anneling T °C	Melting Temp (°C)
MMP2	for 5'-ataacctggatgccgtcgt-3' rev 5'-aggcacccttgaagaagtagc-3'	UPL #70	63 nt	2	60	95
MMP9	for 5'-atccggcacctctatggtc-3' rev 5'-ctgaggggtggacagtgg-3'	UPL #43,#6	121 nt	2	60	95
S1P1	for 5'-ggctggaactgcatcagtgcg-3' rev 5′-gagcagcgccacattctcagagc-3′		223 bp	4	60	89–90
S1P2	for 5′-ccgaaacagcaagttccact-3′ rev 5′-ccaggaggctgaagacagag-3′		197 bp	2	61	90
S1P3	for 5′-aaggctcagtggttcatcgt-3′ rev 5′-gctattgttgctgctgcttg-3′		201bp	2	61	92–93
S1P4	for 5′-ccttcagcctgctcttcact-3′ rev 5′-aagaggatgtagcgcttgga-3′		223 bp	4	64	94
S1P5	for 5′-aggacttcgcttttgctctg-3′ rev 5′-tctagaatccacggggtctg-3′		201bp	3	59	87
PBGD	for 5′-tccaagcggagccatgtctg-3′ rev 5′-agaatcttgtcccctgtggtgga-3′		204 bp	3	62	89
GAPDH	for 5'-gttcgacagtcagccgcatc-3' rev 5'-ggaatttgccatgggtgga-3'	5'- accaggcgcccaatacgaccaa -3'	229 nt	2	60	95
HPRT	for 5′-tgtaatgaccagtcaacaggg-3′ rev 5′-tggcttatatccaacacttcg-3′			2	60	94

Quantitative real-time PCR (qPCR)—Real-time PCR assays for MMP2 and MMP9 were designed with the Universal Probe Library (UPL) Assay Design Center (www.rocheapplied-science.com). MMP2 and MMP9 primers were purchased from TAG Copenhagen (Copenhagen, Denmark) and UPL probes from Roche (Basel, Switzerland). MMP primers and the probe for the reference gene GAPDH were from Oligomer (Helsinki, Finland). For MMP2 qPCR, reaction mixtures were prepared with KAPA Probe Fast Master Mix (KAPA Biosystems; Boston, MA) and qRT-PCR was performed with the StepOnePlus Real-Time PCR System (Applied Biosystems; Waltham, MA) using the relative standard curve method. The MMP9 qPCR reaction mixtures for FTC-133 cells were prepared with PowerUp ^TM^ SYBR^TM^ Green Master Mix according to the manufacturer’s instructions (Applied biosystems, Thermo Fisher Scientific, MA, USA) and qRT-PCR was performed with the StepOnePlus Real-Time PCR System (Applied Biosystems; Waltham, MA) using the relative standard curve method. For MMP9, porphobilinogen deaminase (PBGD) and for MMP2, glyceraldehyde-3-phosphate dehydrogenase (GAPDH) was used as reference genes.

### Transient transfections

4 000,000 cells were pelleted and suspended in 200 μl OptiMem. siRNA (2 μM) was suspended in 200 μl OptiMem. The two suspensions were mixed, incubated for 15 min at room temperature and transferred into an electroporation cuvette (0.4 cm). The cells were electroporated at 240 V and 500 μF. The electroporated cells were transferred to 100-mm cell culture plates and used in experiments 48 h after transfection. A non-targeting siRNA was used as negative control.

### Invasion assays

Invasion assays were performed as described elsewhere [10]. Cells were grown in 0.2% FAF-BSA-containing serum-free medium (SFM) overnight before the start of an experiment. In some experiments, cells were pre-incubated with JTE-013 (10 μM, 1 h), VPC 23019 (1 μM, 1 h), C3-Transferase (100 ng/ml, 4 h), Y-27632 (10 μM, 1 h), SB-3CT (10 μM, 1 h) or ALLN (50 μM, 1 h). The inhibitors were present in both the upper and lower chambers throughout the experiment. The cells were stimulated with S1P and allowed to migrate towards 5% lipid-stripped FBS (LS-FBS) for the time points indicated and then non-migrated cells were removed with a cotton swab. The migrated cells were fixed in 2% paraformaldehyde for 10 min and then stained with 0.1% crystal violet in 20% methanol for 5 min. The membranes were washed with PBS and water and allowed to dry overnight. The cells were counted at 40X magnification in eight to ten fields in a straight line bisecting the membrane. Images of invasion inserts were captured using Leica EC3 camera (Leica Microsystems, Switzerland Ltd., CH, Switzerland) and with 20X magnification objective on Leica microscope (Leica Microsystems CMS GmbH, Wetzlar, Germany).

### Wound healing assays

Cell were grown to 90% confluency. On the day prior to an experiment, the medium was changed to SFM. The next day, fresh SFM was added to the plates. 0.5 μg/ml mitomycin C was added to each plate to inhibit cell proliferation. The monolayer of cells was scratched with a micropipette tip (200 μL). The cells were treated with 100 nM S1P while vehicle was added in the control plates. The images were taken immediately (0 h) and after 24 h with a Leica microsystem camera and framework software (Leica Microsystems; Wetzlar, Germany). The distance traveled by the cells to close the wound was measured. The data is presented as % wound.

### Zymography assays

Cells were grown on 35-mm plates to 90% confluency. The medium was changed to SFM overnight. The next day, fresh SFM was added to the plates and the cells were stimulated with S1P or vehicle. After 6 h, the medium was collected from each plate. Equal volumes of medium were mixed with loading buffer (0.1 M Tris-phosphate buffer, pH 6.8, containing 20% glycerol, 6% SDS, and 0.04% bromophenol blue). The samples were electrophoresed with 10% SDS gels containing gelatin (2.65 mg/ml). Next, the gels were incubated in Zymo buffer (50 mM Tris-HCl containing 2.5% Tween 80 and 0.02% NaN_3_, pH 7.5) for 30 min. The gels were then incubated in Zymo I buffer containing 1 μM ZnCl_2_ and 5 mM CaCl_2_ for 30 min. For gelatinolytic activity, gels were incubated at 37C° overnight in buffer containing 50 mM Tris-HCl, 5 mM CaCl_2_, 1 μM ZnCl_2_, and 0.02% NaN_3_ (pH 7.5). The gels were visualized under UV light for protein ladder marker and after that the gels were stained with Coomassie Blue R250 for 1–2 h. The gelatinolytic activity was visualized as clear bands against blue background on stained gels. The bands were quantified with the Image J program. The data were normalized with the respective total protein concentrations of the cells on the culture plates.

### Western blotting

The cells were grown to 90% confluency. The medium was changed to SFM overnight. On the day of the experiment, fresh SFM was added to the plates and the cells were stimulated with S1P for 6 h. In some experiments, the cells were pre-incubated with the S1P_2_ inhibitor JTE—013 (10 μM, 1 h), the S1P_1,3_ inhibitor VPC—23019 (1μM, 1 h), MMP2/9 inhibitor SB-3CT (10 μM, 1 h), calpain inhibitor ALLN (50 μM, 1 h), the ROCK inhibitor Y27632 (10 μM, 1 h) or with Rho inhibitor C3-transferase (100 ng/ml, 4 h). After collection of medium for secretion experiments, the cells were washed with ice cold PBS and lysed in lysis buffer (10 mM Tris, pH 7.7, 150 mM NaCl, 7 mM EDTA, 0.5% NP-40). Cell lysates were centrifuged at 13,000 rpm for 15 min at 4C°. The supernatants were collected and their protein concentration was measured with a BCA protein assay kit. Laemmli sample buffer was added to all samples and western blotting was performed as described previously [6]. The primary antibodies used were anti-MMP2 (1:500), anti-MMP9 (1:500), Hsc70 (1:5000) and β-actin (1:1000). The secondary antibodies used were peroxide-conjugated goat anti- rabbit (1:3000), anti-mouse (1:3000) and anti-rat (1:3000). β-actin and Hsc70 were used as loading control in MMP and S1P receptor expression experiments. However, in MMP secretion experiments, the amounts of secreted MMPs were normalized against total protein concentrations of the cells on the plates.

### Calpain activity assays

2 000,000 cells were grown on 100-mm plates. The cells were serum-starved overnight and treated with 100 nM S1P for 6 h. The cells were detached and washed three times with PBS. Thereafter, calpain activity assays were processed according to the manufacturer’s instructions. Fluorescence was measured using a Hidex sense microplate reader instrument (HIDEX Corp, Turku, Finland) with excitation at 400 nm and emission at 505 nm. The protein concentration was kept at 60 mg/ml for each sample.

### Rho activation assays

50,000 cells were plated on 35-mm plates, grown until they reached 50% confluency, and then serum-starved for 24 h. The cells were treated with 100 nM S1P for 0, 1, 3, 6 and 12 min and the lysates were made, aliquoted for protein concentration measurement, snap-frozen immediately after harvesting and clarified as directed in the user’s manual. Protein concentration was measured using the BCA kit. The protein concentration used for assays was kept at 0.5 mg/ml in each sample. Rho activation was measured with the G-LISA ^TM^ Rho activation assay kit (Cytoskeleton, Inc.; Denver, CO, USA) according to the manufacturer’s instructions.

### Statistics

Results are presented as mean ± SEM from at least three independent measurements. Student’s *t*-test was applied to the data when two means were compared. One-way ANOVA and Bonferroni’s *post hoc* test was used when three or more means were compared. The GraphPad Prism 5 software (GraphPad Software Inc.; San Diego, CA) was used for the statistical analyses. P-values < 0.05 were considered statistically significant.

## Results

### S1P inhibits expression, secretion and activity of MMP2 and MMP9 in C643 cells

**Fig 1:**
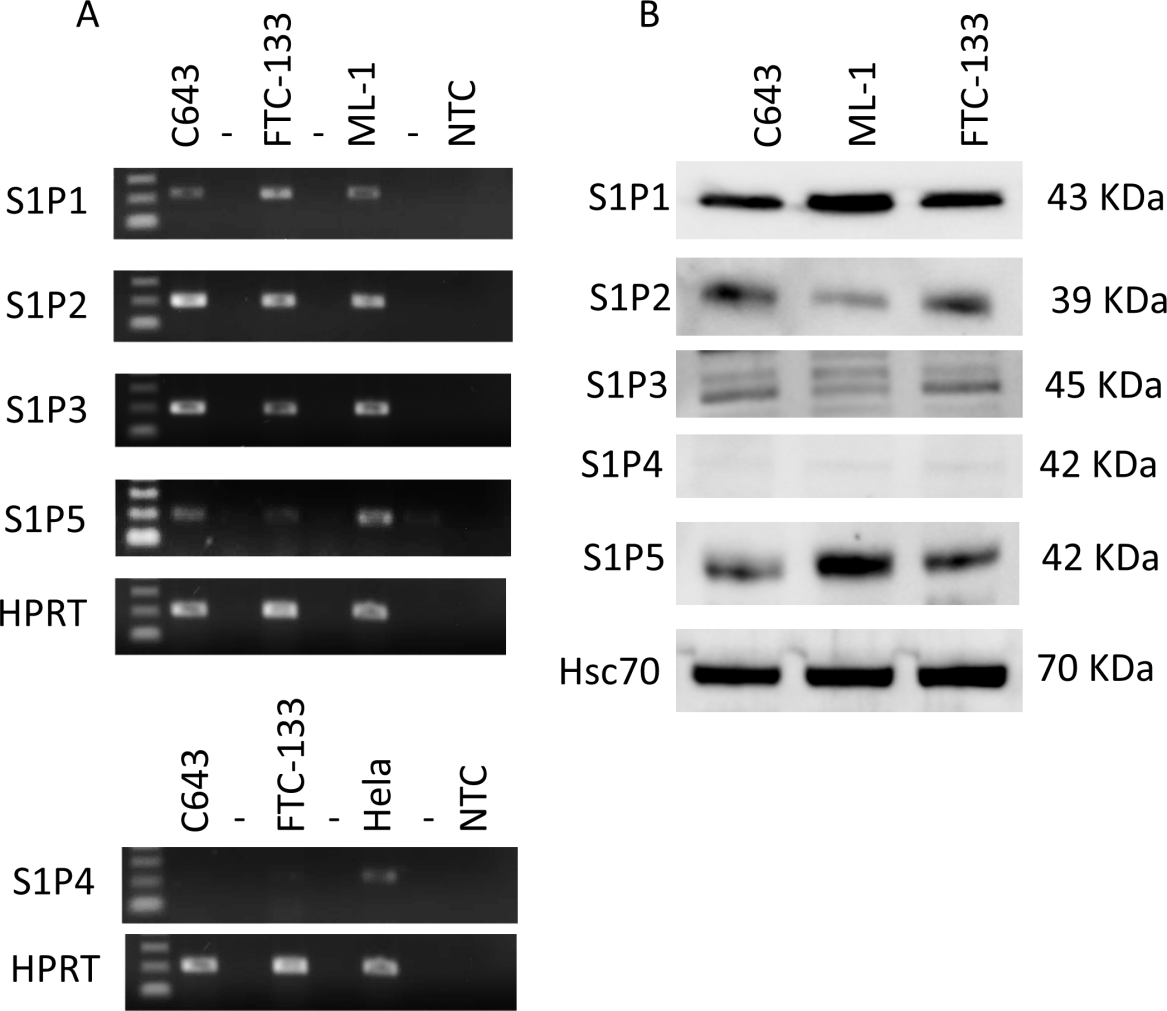
Expression of S1P receptors in human thyroid cancer C643 and FTC-133 cells. (A) The mRNA expression of S1P_1-5_. Representative agarose gels showing the PCR products stained with ethidium bromide. ML-1 cDNA was used as positive control for S1P_1-3_ and S1P_5_. Hela cDNA was used as positive control for S1P_4_. HPRT was used as control reference gene. (B) Representative western blots showing the expression of S1P_1-5_ on protein level. Hsc70 was used as loading control. Each gel or blot is a representative of at least three independent experiments.

Previously, we have shown that S1P (100 nM, 6 h) inhibits migration and invasion of human anaplastic thyroid cancer C643 and follicular thyroid cancer FTC-133 cells towards lipid-stripped serum. This attenuated migration is mediated by S1P_2_ and the Rho-ROCK pathway [10]. Interestingly, S1P promotes migration and invasion in human follicular thyroid cancer ML-1 cells [6–8,21] and increases the secretion and activity of MMP2 and MMP9 through S1P_1,3_ [19]. This effect on MMP2/9, at least in part, mediates S1P-evoked invasion of these cells [19]. As can be seen in (Fig 1A and 1B): S1P_1-3_ and S1P_5_ are expressed in C643 and FTC-133 cells. Furthermore, neither of these cell line expressed S1P_4_. Despite the same S1P receptor expression profile, S1P promotes migration in ML-1 cells [8] and inhibits migration in C643 and FTC-133 cells [10].

**Fig 2:**
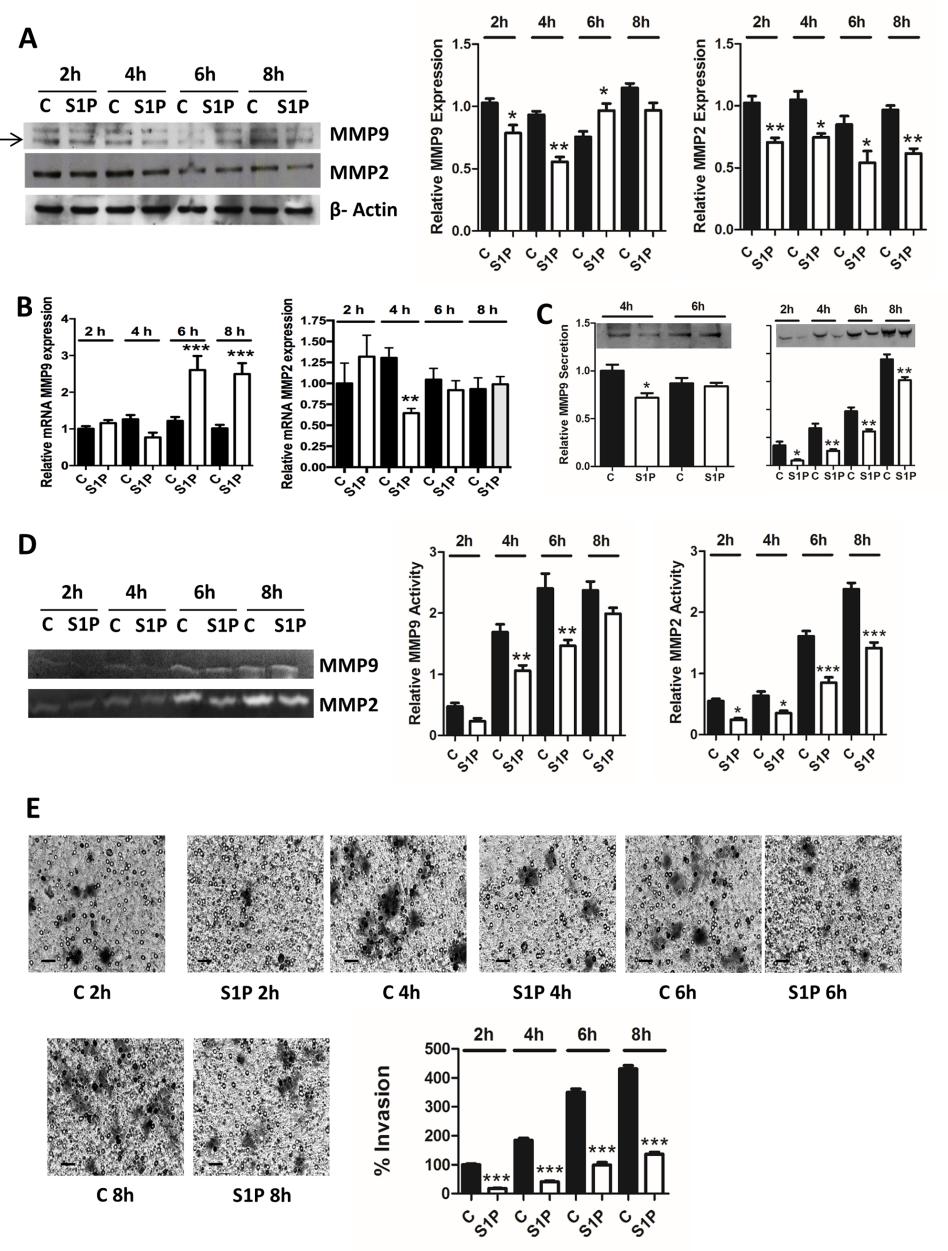
S1P inhibits invasion and affects expression, secretion and activity of MMP2 and MMP9 in C643 cells. (A) Cells were treated with S1P (100 nM for 2 h, 4 h 6 h and 8 h). The representative western blots show the expression of MMP2 and MMP9. β-Actin was used as loading control and the normalized data are presented in the graphs. (B) Cells were treated with S1P (100 nM for 2 h, 4 h, 6 h and 8 h). MMP2 and MMP9 mRNA expression was measured with quantitative real-time PCR. (C) Cells were treated with S1P (100 nM for 2 h, 4 h, 6 h and 8 h). The representative western blots show the secretion of MMP2 and MMP9. (D) Cells were treated with S1P (100 nM for 2 h, 4 h, 6 h and 8 h). The representative zymography blot shows the activity of MMP2 and MMP9. The data were normalized to the protein content on the plates and are presented in respective graphs. (E) C643 cells were allowed to invade through collagen IV towards 5% lipid-stripped FBS in the presence or absence of S1P (100 nM for 2 h, 4 h, 6 h and 8 h). Representative images of invasion inserts are shown. The scale bar is 50 μm. The normalized data in the graphs are the mean ± S.E.M, n = 3–6. Asterisks (*) denote the statistically significant differences compared with respective control. Data were analyzed with one-way ANOVA and Bonferroni’s post hoc test (* *P* < 0.05; ** *P* < 0.01; *** *P* <0.001).

In the present study, we have investigated the role of MMP2 and MMP9 in C643 cells. Our results show for the first time that stimulation with S1P decreased expression, secretion, and activity of MMP2 at 2 h, 4 h, 6 h and 8 h (Fig 2A, 2C and 2D). Stimulation with S1P also decreased the expression of MMP9 at 2 h and 4h, secretion at 4 h and activity at 2 h, 4 h, and 6 h (Fig 2A, 2C and 2D). Interestingly, S1P significantly increased the expression, but not the secretion, of MMP9 at 6 h (Fig 2A and 2C). We next determined the effect of S1P on MMP2 and MMP9 mRNA expression after 2 h, 4 h, 6 h and 8 h of S1P treatment, and as can be seen in Fig 2B, S1P significantly decreased MMP2 mRNA expression at 4 h but no effect was observed in other time points. S1P had no effect on MMP9 mRNA expression at 2 h and 4 h but caused a marked increase in MMP9 mRNA at 6h and 8h.

**Fig 3:**
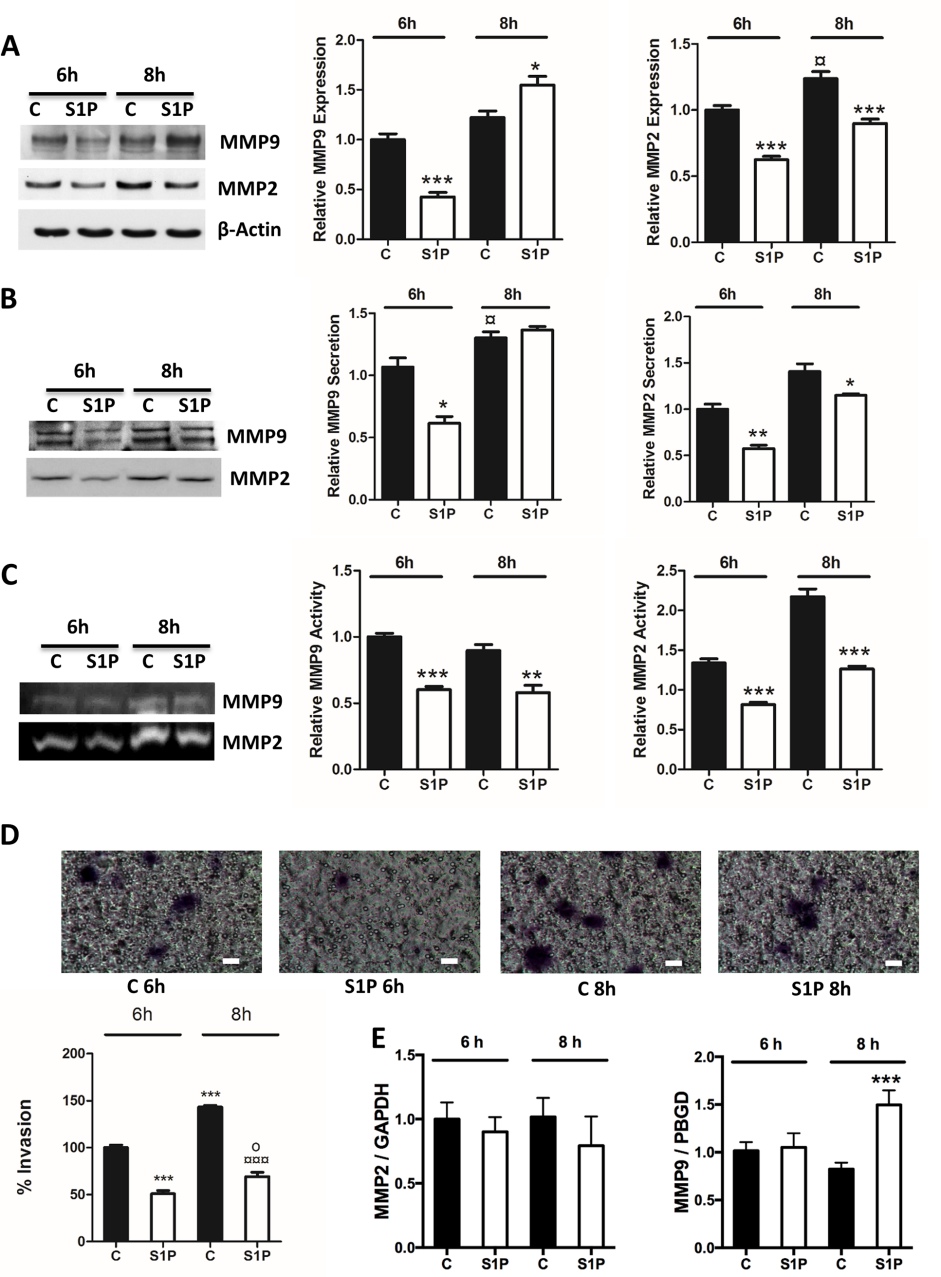
S1P inhibits invasion and affects expression, secretion and activity of MMP2 and MMP9 in FTC-133 cells. (A) Cells were treated with S1P (100 nM for 6 h and 8 h). The representative western blots show the expression of MMP2 and MMP9. β-Actin was used as loading control and the normalized data are presented in the graphs. (B) Cells were treated with S1P (100 nM for 6 h and 8 h). The representative western blots show the secretion of MMP2 and MMP9 and the normalized data are presented in the graphs. (C) Cells were treated with S1P (100 nM for 6 h and 8 h). The representative zymography blot shows the activity of MMP2 and MMP9. The data were normalized to the protein content on the plates and are presented in respective graphs. (D) C643 cells were allowed to invade through collagen IV towards 5% lipid-stripped FBS in the presence or absence of S1P (100 nM for 6 h and 8 h). Representative images of invasion inserts are shown. The scale bar is 50 μm. (E) MMP2 and MMP9 mRNA expression was measured with quantitative real-time PCR and normalized to GAPDH and PBGD, respectively. The normalized data in the graphs are the mean ± S.E.M, n = 3–4. Asterisks (*) denote the statistically significant differences compared with the respective control, (¤) denotes the statistically significant differences between controls and (o) denotes the statistical difference between S1P treatments. Data were analyzed with one-way ANOVA and Bonferroni’s post hoc test (* *P* < 0.05; ** *P* < 0.01; *** *P* <0.001; ¤ *P* < 0.05; o *P* < 0.05).

To correlate the S1P-evoked response on MMP2 and -9 with the invasion of C643, we measured the effect of short S1P stimulations on C643 invasion. Our results show that S1P potently attenuates invasion at 2 h, 4 h, 6 h, and 8 h of stimulation (Fig 2E). In addition, S1P also decreased the expression, secretion and activity of MMP2 at 6 h and 8 h in thyroid follicular cancer FTC-133 cells (Fig 3A, 3B and 3C). Furthermore, at these time points, S1P potently attenuated the invasion of these cells (Fig 3D). However, S1P, has no effect on MMP2 mRNA expression in 6 h and 8h but significantly increased MMP9 mRNA at 8h (Fig 3E). We observed the same biphasic response of S1P on MMP9 in these cells as we observed in C643 cells: after 6 h of incubation, S1P decreased the expression and secretion of MMP9, whereas at 8 h of incubation, the expression, but not the secretion, was increased. However, S1P decreased the activity of MMP9 at both 6 h and 8 h (Fig 3C).

### S1P-induced inhibition of expression, secretion and activity of MMP2 is mediated by S1P_2_

**Fig 4:**
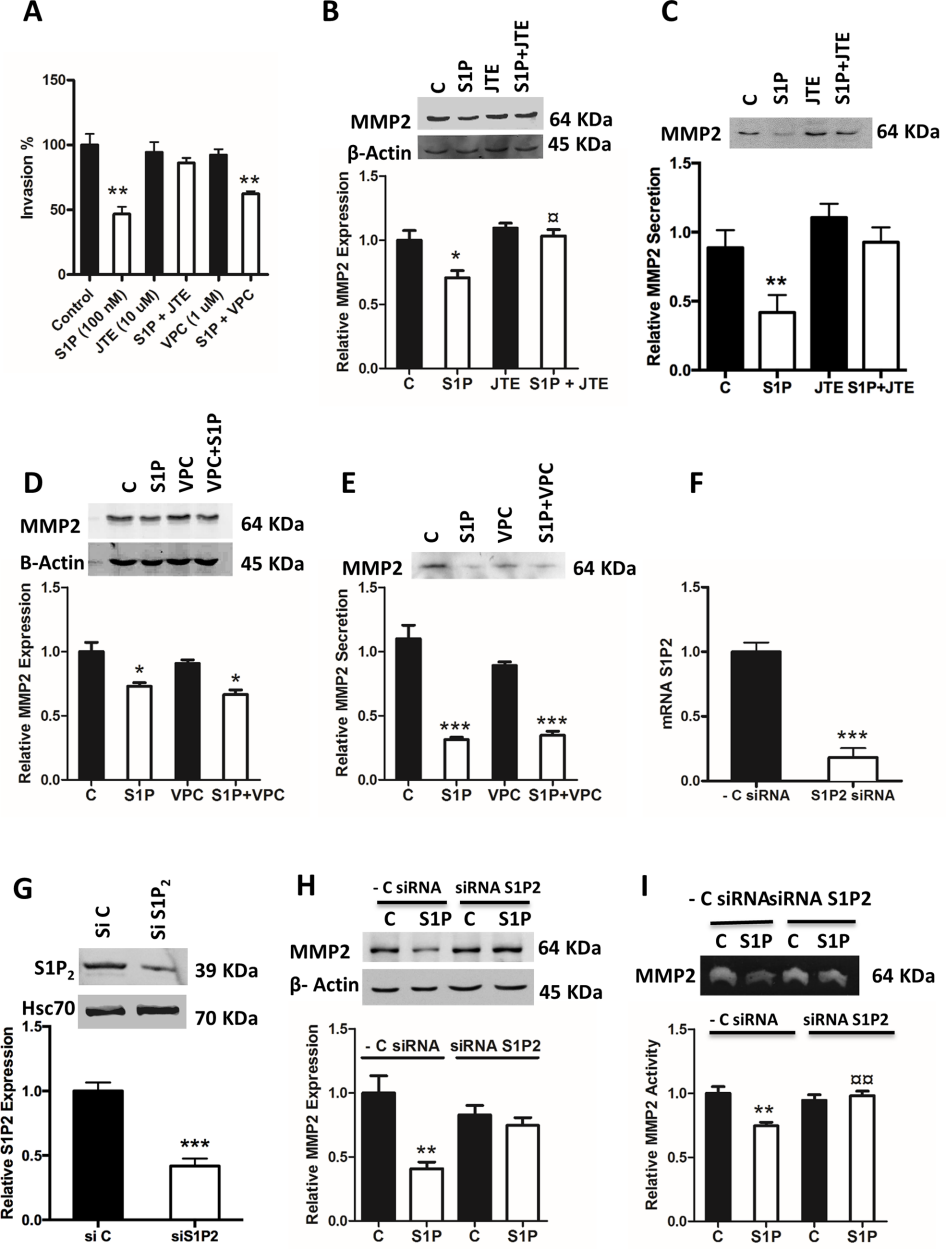
S1P-evoked inhibition of invasion and the expression, secretion and activity of MMP2 is mediated through S1P_2_. (A) C643 cells were pre-incubated with S1P_2_ antagonist JTE-013 (10 μM for 1 h) or S1P_1,3_ inhibitor VPC23019 (1 μM for 1 h) and allowed to invade through collagen IV towards 5% lipid-stripped FBS in the presence or absence of 100 nM S1P for 6 h. (B) C643 cells were treated with S1P_2_ antagonist JTE-013 (10 μM for 1 h) and then stimulated with S1P (100 nM for 6 h). A representative western blot is shown. β-Actin was used as loading control and the normalized data are presented in the graph. (C) C643 cells were treated with the S1P_2_ antagonist JTE-013 (10 μM for 1 h) and then stimulated with S1P (100 nM for 6 h), and the medium was collected. The representative western blot shows the secretion of MMP2. The data were normalized to the protein content on the plates and are presented in the graph. (D). C643 cells were treated with S1P_1,3_ inhibitor VPC23019 (1 μM for 1 h) and then stimulated with S1P (100 nM for 6 h). A representative western blot is shown. β-Actin was used as loading control and the normalized data are presented in graph. (E) C643 cells were treated with S1P_1,3_ inhibitor VPC23019 (1 μM for 1 h) and then stimulated with S1P (100 nM for 6 h), and the medium was collected. The representative western blot shows the secretion of MMP2. The data were normalized to the protein content on the plates and are presented in the graph. (F and G) C643 cells after 48 h transfection with S1P_2_ siRNA. The normalized graph shows the efficient knockdown of S1P_2_ on mRNA level and the representative western blot shows the knockdown of S1P2 on protein level. (H and I) C643 cells were transfected with scrambled control siRNA or S1P_2_ siRNA and stimulated with 100 nM S1P for 6 h. (H) A representative western blot shows the expression of MMP2. β-Actin was used as loading control and the normalized data are presented in graph. (I) A representative zymography blot shows the activity of MMP2. The data were normalized to the protein content on the plates. The normalized results in the graphs are the mean ± S.E.M, n = 3–4. Asterisks (*) denote the statistically significant differences compared with respective control. (¤) indicates comparisons between S1P effects. Data were analyzed with one-way ANOVA and Bonferroni’s post hoc test (* *P* < 0.05; ** *P* < 0.01; *** *P* <0.001; ¤ *P* < 0.05; ¤¤ *P* < 0.01).

Previously, we have shown that C643 cells express S1P_1-3_ [10]. First, we investigated the effect of S1P on the invasion of C643 cells. In C643 cells pre-treated with the S1P_1,3_ antagonist VPC-23019 (1 μM, 1 h) or the S1P_2_-antagonist JTE-013 (10 μM, 1 h), and then stimulated with S1P for 6 h, showed that the S1P-evoked inhibition of C643 invasion was mediated by S1P_2_ and not by S1P_1,3_ (Fig 4A), as was also shown previously [10]. We next investigated which S1P receptor was involved in the S1P-induced attenuation of the expression, secretion and activity of MMP2 and -9. For this, the C643 cells were again pre-incubated with either VPC-23019 or JTE-013 and then stimulated with S1P for 6 h. The results showed that the S1P-induced inhibition of MMP2 expression and secretion was abolished by JTE-013 (Fig 4B and 4C), whereas VPC-23019 had no effect on S1P-induced inhibition of MMP2 expression and secretion (Fig 4D and 4E). To confirm these findings, the C643 cells were transfected with S1P_2_ siRNA. Both the mRNA and protein expression of S1P_2_ was efficiently downregulated (Fig 4F and 4G). As can be seen in Fig 4H and 4I, the S1P-evoked attenuation of MMP2 expression and activity was abolished by S1P_2_ knockdown. Surprisingly, neither VPC-23019 nor JTE-013 had an effect on the S1P-evoked effects on MMP9 (data not shown). Thus, the S1P-induced inhibition of expression and activity of MMP2 is mediated by S1P_2_.

### MMP2 and MMP9 mediate C643 invasion

**Fig 5:**
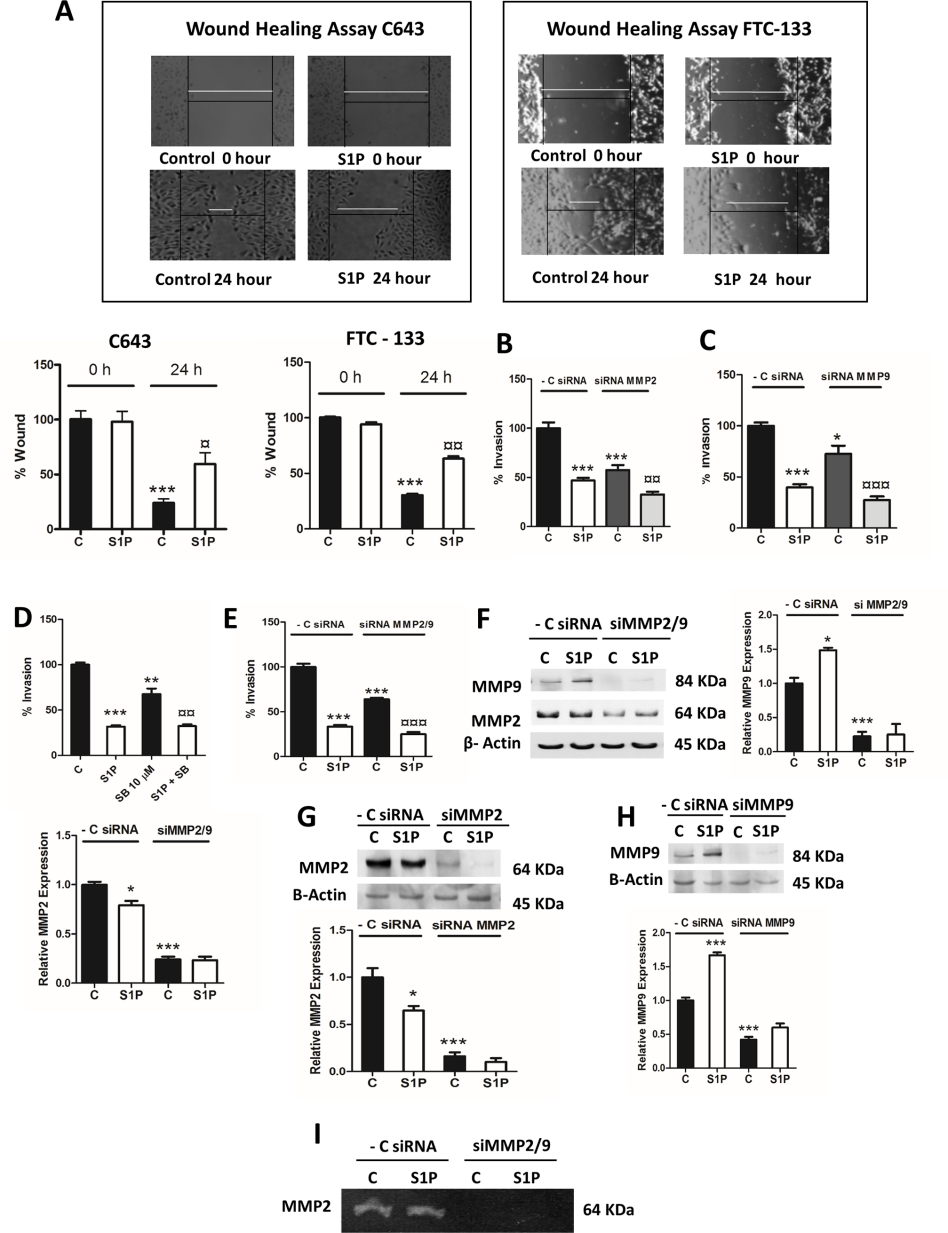
MMP2 and MMP9 regulate C643 invasion. (A) Wound healing assay for C643 and FTC-133 cell migration. Images were taken immediately after wound scratch at 0 h and after 24 h. The cells were stimulated with vehicle or 100 nM S1P. The images are representative of three separate experiments. The data are presented as % wound in respective graphs. (B and C) C643 cells were transfected with negative control siRNA, MMP2 siRNA or with MMP9 siRNA. After 48 hours of transfection, the cells were allowed to invade through collagen IV towards 5% lipid-stripped FBS for 6 h. (D) C643 cells were pre-incubated with the MMP2/9 inhibitor SB (10 μM, 1 h) and were allowed to invade towards 5% lipid-stripped FBS for 6 hours in the presence or absence of 100 nM S1P. (E) C643 cells were transfected with negative control siRNA or with siRNA for MMP2 and -9. After 48 hours of transfection, the cells were allowed to invade towards 5% lipid-stripped FBS for 6 hours in the presence or absence of 100 nM S1P. (F) C643 cells were transfected with negative control siRNA or with siRNA for MMP2 and -9. After 48 hours of transfection, the cells were stimulated with S1P for 6 hours and the expression of MMP2 and -9 was measured. A representative western blot is shown. β-Actin was used as loading control, and the normalized results are presented in the graphs. (I) As in (F), but the medium was collected and the activity of MMP2 measured using the zymography assay. The data were normalized to the protein content on the plates. (G and H) C643 cells were transfected with negative control siRNA, with MMP2 siRNA or MMP9 siRNA. After 48 hours of transfection, the cells were stimulated with 100 nM S1P for 6 hours and the expression of MMP2 and -9 was measured. Representative western blots are shown. β-Actin was used as loading control, and the normalized results are presented in the graphs. The normalized results in the graphs are the mean ±S.E.M, n = 3. Asterisks (*) denote the statistically significant differences compared with respective control. (¤) indicates comparisons between S1P effects. Data were analyzed with one-way ANOVA and Bonferroni’s post hoc test (* *P* < 0.05; ** *P* < 0.01; *** *P* <0.001; ¤ *P* < 0.05; ¤¤ *P* < 0.01; ¤¤¤ *P* <0.001).

MMP2 and MMP9 are gelatinases which use collagen IV as their main substrate and regulate thyroid cancer cell invasion [13,17,18]. Recently, we have shown that blocking MMP2 and MMP9 attenuated S1P-evoked follicular ML-1 thyroid cancer cell invasion [19]. As shown above, S1P attenuated the invasion of both C643 and FTC-133 cells at 6 h and 8h (Figs 2E and 3D). S1P also attenuated the wound healing of both C643 and FTC-133 cells (Fig 5A). This decrease in migration and invasion is not due to a decrease in proliferation, as S1P was without an effect on C643 and FTC-133 proliferation [10]. To verify the importance of MMP2 and MMP9 in C643 cell invasion, we down-regulated MMP2 and -9 with siRNA. In C643 cells transfected with siRNA against MMP2 or MMP9, basal invasion was significantly attenuated compared with control siRNA-transfected cells. However, S1P was able to significantly further inhibit invasion (Fig 5B and 5C). Next, we used either the MMP2/9 inhibitor SB-C3T (10 μM, 1 h) or double-transfection of MMP2/9 siRNA and found both treatments to significantly attenuate basal invasion of C643 cells (Fig 5D and 5E). In these experiments, S1P was again able to further attenuate invasion, showing that also other mechanisms are involved in the S1P-evoked inhibition of invasion (for a review, see [9]). The siRNA transfections caused a 60–80% knockdown of MMP2 and MMP9 expression, and S1P did not modulate their expression further (Fig 5F–5H). Knockdown of MMP2/9 also completely abolished MMP2 activity (Fig 5I). Taken together, the results clearly show that MMP2 and MMP9 participate in regulating C643 cell invasion.

### S1P regulates calpain activity via S1P_2_ and calpain mediates S1P-induced effects on MMP2/9

**Fig 6:**
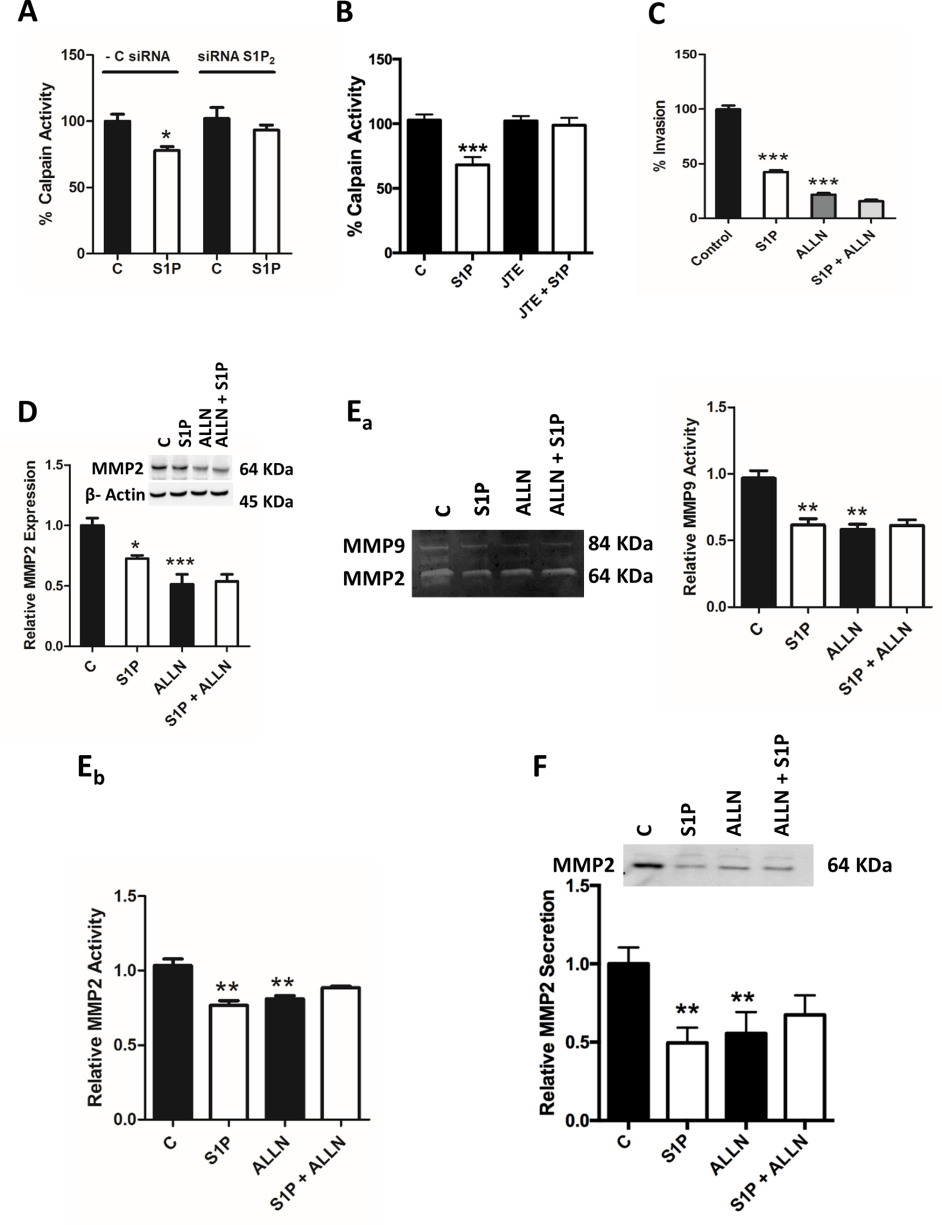
Calpains participate in the expression, activity and secretion of MMP2 and MMP9. (A) C643 cells were transfected with negative control or with S1P_2_ siRNA for 48 h, and then stimulated with 100 nM S1P for 6 h, and the calpain activity was measured. (B) C643 cells were treated with the S1P_2_ antagonist JTE-013 (10 μM for 1 h) and then stimulated with S1P (100 nM for 6 h), and the calpain activity was measured. (C) C643 cells were pre-incubated with calpain inhibitor ALLN (50 μM for 1 h) and allowed to invade through collagen IV towards 5% lipid-stripped FBS in the presence or absence of 100 nM S1P for 6 h. The data are presented in the graph. (D) C643 cells were pre-incubated with calpain inhibitor ALLN (50 μM for 1 h) and then stimulated with 100 nM S1P for 6 hours. The expression of MMP2 was then determined. Representative western blots are shown. β-Actin was used as loading control, and the normalized data are presented in respective graphs. (Ea,b) C643 cells were pre-incubated with calpain inhibitor ALLN (50 μM for 1 h) and then stimulated with S1P for 6 h. The medium was collected and the zymography assay was performed. A representative zymography blot is shown. The data were normalized to the protein content on the plates and are presented as graphs. (F) C643 cells were pre-incubated with calpain inhibitor ALLN (50 μM for 1 h) and then stimulated with S1P for 6 hours. The medium was collected and the secreted MMP2 was measured. A representative western blot is shown. The data were normalized to the protein content on the plates. The normalized results in the graphs are the mean ±S.E.M, n = 3–6. Asterisks (*) denote the statistically significant differences compared with respective control. Data were analyzed with one-way ANOVA and Bonferroni’s post hoc test (* *P* < 0.05; ** *P* < 0.01; *** *P* <0.001).

Calpains are calcium-dependent endopeptidases, and in addition to many other important functions in the cell, they cleave pro-MMP2/9 to convert them into active MMP2/9. Furthermore, calpains are regulated by S1P [22]. We have recently shown that calpains are of importance in the S1P-evoked secretion of MMP2/9 in follicular thyroid cancer ML-1 cells [19]. We thus investigated the role of calpains in C643 cells. Stimulating the cells with S1P (100 nM, 6 h) attenuated calpain activity and knockdown of S1P_2_ with siRNA or JTE-013 abolished this effect (Fig 6A and 6B). Next, we investigated if calpains are involved in invasion of C643 cells. As can be seen in Fig 6C, basal invasion was significantly attenuated when C643 cells were pre-incubated with the calpain inhibitor ALLN (50 μM, 1 h). However, S1P could still further decrease invasion. To determine whether calpain inhibition affects MMP2/9 expression, secretion and activity, C643 cells were pre-incubated with ALLN (50 μM, 1 h) and then stimulated with S1P (100 nM, 6 h) or vehicle. In these experiments, the basal expression of MMP2 and basal activity of MMP2 and MMP9 were significantly attenuated by ALLN (Fig 6D and 6E_a-b_). ALLN also prevented the S1P-induced inhibition of MMP2 and -9 activity (Fig 6E_a-b_). In addition, in C643 cells treated with ALLN the secretion of MMP2 was strongly attenuated and S1P was not able to decrease it further (Fig 6F). These results show that S1P through S1P_2_ inhibits calpain and that calpain in turn mediates the S1P-induced effects on MMP2/9.

### The Rho-ROCK pathway mediates S1P-induced inhibition of MMP2

**Fig 7:**
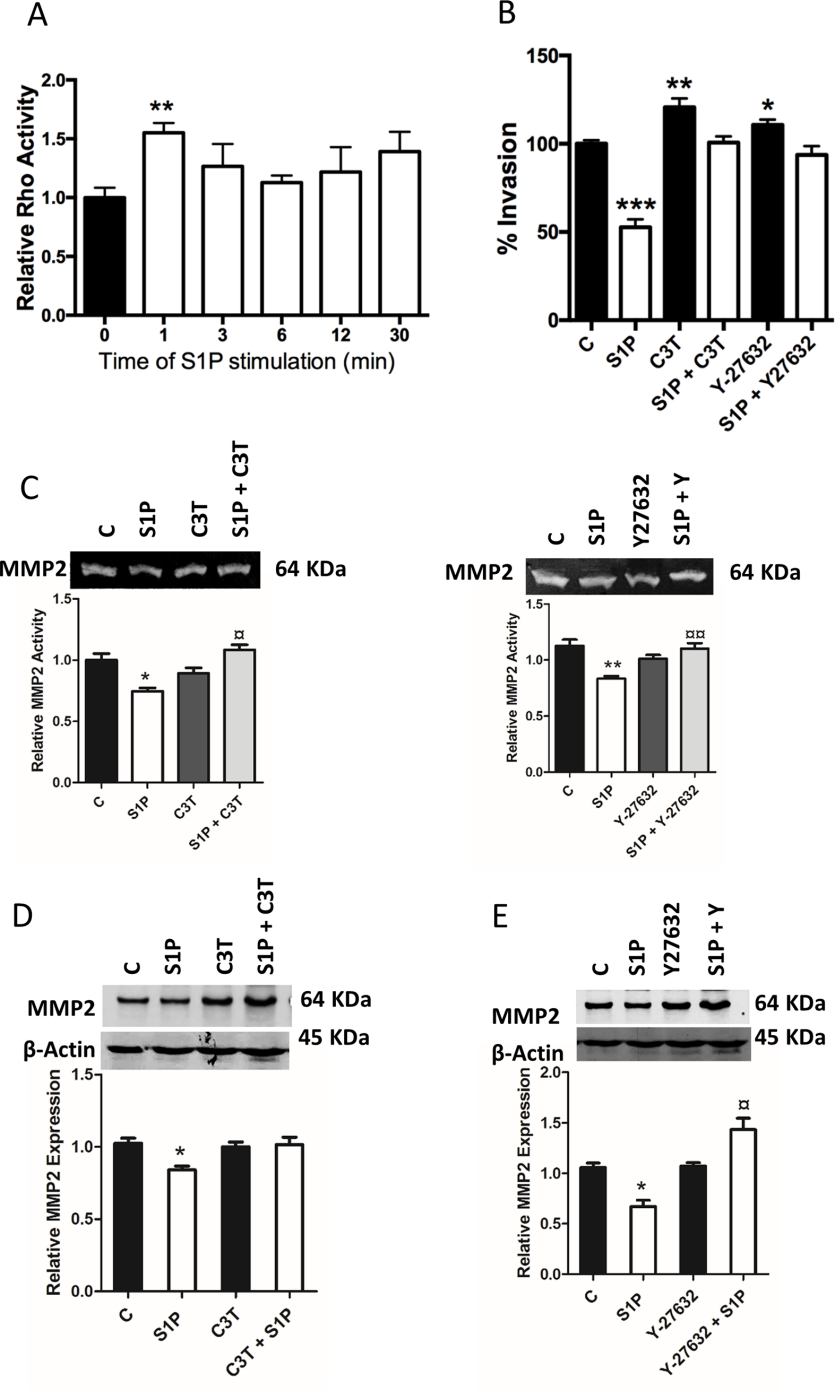
S1P-evoked inhibition of MMP2 is mediated through the Rho-ROCK pathway. (A) Time-dependent effect of S1P on Rho activity in C643 cells. (B) Stimulatory effect of the Rho inhibitor C3-transferase or the ROCK inhibitor Y-27632 on C643 cell invasion. (C) C3-transferase or Y-27632 attenuated the S1P-evoked inhibition of MMP2 activity. (D and E) Lack of an effect of pretreatment with C3-transferase or Y-27632 on the expression of MMP2 but S1P-evoked-attenuation of MMP2 expression was abolished. The representative blots are shown. β-Actin was used as loading control, and the normalized results are presented in graphs. The data in the graphs are the mean ±S.E.M, n = 3. Asterisks (*) denote the statistically significant differences compared with respective control. (¤) indicates comparisons between S1P effects. Data were analyzed with one-way ANOVA and Bonferroni’s post hoc test (* *P* < 0.05; ** *P* < 0.01; *** *P* <0.001; ¤ *P* < 0.05; ¤¤ *P* < 0.01).

Previous studies have shown that S1P through S1P_2_ activates Rho, and that Rho in turn inhibits cell motility through activation of ROCK and inhibition of Rac [10,23,24]. Also, we have recently shown that the S1P-induced inhibition of C643 migration is mediated by the intermediate filament protein vimentin whose phosphorylation S1P induces through S1P_2_ and ROCK [25]. In Fig 7A we show that S1P, as expected, transiently increased Rho activity in C643 cells. Pre-incubation of these cells with the Rho inhibitor C3-transferase (100 ng/ml, 4 h) or with the ROCK inhibitor Y-27632 (10 μM, 1 h) resulted in a significant increase in basal invasion. Furthermore, the S1P-induced inhibition of invasion was abolished (Fig 7B). Zymography experiments revealed that inhibition of Rho or ROCK had no significant effect on basal MMP2 activity but that the S1P-evoked decrease in MMP2 activity was abolished (Fig 7C). The Rho inhibitor had no significant effect on basal MMP2 expression. However, Rho inhibition did prevent the S1P-induced decrease in MMP2 expression (Fig 7D). When the cells were treated with the ROCK inhibitor, there was again no effect on the basal expression of MMP2, but S1P significantly increased the expression of MMP2 instead of decreasing it (Fig 7E). Taken together, these results suggest that S1P decreases MMP2 expression and activity through the S1P_2_-Rho-ROCK pathway.

## Discussion

S1P modulates cell invasion and migration of cancer cells, including thyroid cancer cells, by activating S1P receptors and associated down-stream signaling pathways. S1P_1,3_ are pro-migratory receptors which strongly couple to G_i_ and activate the migratory pathway [3–5,26,27], whereas S1P_2_ usually functions as an anti-migratory receptor that couples strongly to G_12/13_ [23,24,27,28] (but see [11]). In the present report, we show that S1P potently attenuates the expression, secretion and activity of MMP2, and to a lesser degree, the secretion and activity of MMP9. This is a novel mechanism by which the anti-migratory receptor S1P_2_ can convey its effects on cells, and is, in part, a mechanism mediating the inhibitory effect of S1P_2_ on invasion.

Matrix metalloproteinases (MMPs) are important regulators of cell invasion in several malignant forms of cancer [13,17]. Previous studies have indicated that MMP2 and -9 enhance invasion of thyroid cancer cells [15,16,18]. We have recently reported that S1P evokes invasion of thyroid follicular ML-1 cancer cells mainly through S1P_1_ [8], and that this effect is, in part, induced by the S1P-evoked secretion and activity of MMP2 and -9 [19]. In the present study, S1P decreased consistently and significantly the expression, secretion and activity of MMP2. In contrast, S1P evoked a biphasic effect on MMP9: first, a decrease in expression, followed by an increase in expression. This finding is in line with a recent report showing that S1P, through activation of S1P_2_, enhances MMP9 expression and secretion [29]. However, in our cells the secretion was transiently decreased, and the activity was attenuated during all investigated time points. This was an interesting finding, considering that the expression of MMP9 was increased after long incubations with S1P. Presently we do not have any explanation for the biphasic effect of S1P on MMP9 or for the decreased activity of the secreted MMP9. One possibility is that the increased expression is a compensatory mechanism, but that the inhibitory effect of S1P on calpain activity rendered MMP9 inactive.

It is interesting to note that the S1P receptor expression profile between the ML-1 and C643 cells is very similar. Although S1P_1,3_ may also have a pro-migratory role in some cell types, it apparently cannot override the inhibitory effect of S1P_2_ in the C643 cells. It is also worth mentioning, that normal primary thyroid cells [6] and the normal thyroid N-Thy-Ori cell line (Asghar and Törnquist, unpublished data) express these receptors, but S1P is unable to either stimulate or inhibit the invasion of these cells.

After observing the surprising time-dependent effects of S1P on the expression, secretion and activity of MMP2 and -9, we were interested in investigating if this would affect the invasive behavior of the cells. We found that S1P potently decreased invasion of the C643 cells at all time points investigated, as was also shown by us previously [10]. To corroborate our observations, we stimulated thyroid follicular FTC-133 cancer cells with S1P. In these cells, we observed similar effects of S1P on invasion and MMP2 expression, and on secretion and activity of both MMP2 and MMP9, as those observed in the C643 cells. We next investigated the importance of MMP2 and –9 *per se* on the invasion of the cells, as these two MMPs are important for thyroid cancer cell invasion [15,16,18,19]. By using siRNA against MMP2 and MMP9, or by inhibiting MMP2/9 pharmacologically, we showed that the basal invasion was significantly attenuated. However, S1P was still able to further decrease the invasion. This suggests that the inhibition of invasion by S1P is not only mediated through inhibition of MMP2 and MMP9, but also through other signaling mechanisms, as has been shown previously [23,24,27,28,30,31]. The situation is quite similar to what we previously saw in follicular thyroid cancer ML-1 cells, where knock-down of MMP2 and -9 only partially inhibited the invasion in response to S1P. This result clearly suggests that not only MMP2 and -9 are important in regulating thyroid cancer cell invasion.

We have earlier reported that S1P inhibited the migration of C643 cells through activation of S1P_2_ [10], and thus wanted to know which S1P receptor was involved in the S1P-evoked attenuation of the MMPs. We investigated MMP2 only, as these results were more consistent. By using either the S1P_2_ antagonist JTE-013 or S1P_2_ siRNA, we showed that the inhibitory effect of S1P on expression and activity of MMP2 was abolished. S1P_1,3_ have been shown to increase the secretion and activity of MMP2 and MMP9 in several cancer cells [13,19]. However, inhibiting S1P_1,3_ was without a significant effect on the S1P-evoked expression or secretion of MMP2. Interestingly, inhibiting S1P_1,3_ or S1P_2_ was without an effect on MMP9 expression and activity, at least at the time points investigated. Thus, contrary to the report by [29], in C643 cells S1P_2_ has an inhibitory effect on at least MMP2 secretion and activity.

Calpains are important in regulating many cellular functions, including cell invasion, by cleaving the inactive pro-MMPs to active MMPs [32,33], and S1P has been shown to regulate calpain activity [22]. In thyroid cancer ML-1 cells, S1P was able to enhance calpain activity and MMP2 and -9 secretion, thus enhancing invasion [19]. We found that in C643 cells, S1P decreased calpain activity through S1P_2_. Inhibiting calpains resulted in a decreased expression and activity of MMP2 and MMP9, as well as decreased secretion of MMP2. These results are in line with previous studies [22,32,34]. Furthermore, the basal invasion was significantly attenuated; however, S1P was still able to slightly, but significantly, further attenuate invasion. This supports our conclusion that MMP2 and -9 are not the only players in S1P-evoked inhibition of invasion of these cells.

S1P_2_ couples to G_12/13_ and activates the small GTPase Rho [31]. On activation, Rho inhibits Rac, resulting in an attenuated invasion and migration. In the present investigation, S1P increased Rho activity transiently. Previously, we have reported that stimulating the cells with S1P decreased Rac activity and that incubating C643 cells with a Rac inhibitor potently decreased migration [10]. As Rac is required for activation of MMP2 [35], our observed activation of Rho (and thus inhibition of Rac) probably, in part, explains the decreased activation of MMP2. Furthermore, pharmacological inhibition of either Rho or ROCK, slightly, but significantly, enhanced invasion of C643 cells. Similar findings were observed in mesenchymal stromal cells, where treatment of cells with either C3-transferese or Y27632 enhanced the migration towards serum [36]. Interestingly, no significant change was, however, observed on the basal expression of MMP2 and -9 in these cells, but the S1P-evoked decrease in the expression and activity of MMP2 was inhibited. Thus, the Rho pathway is important for the regulation of at least MMP2.

We conclude that the S1P regulates the expression, secretion and activity of MMP2 and MMP9. This, in part, attenuates C643 cancer cell invasion. Our data introduces a new mechanism by which S1P, through activation of S1P_2_, can attenuate invasion and migration of cancer cells. Our results thus broaden the palette by which S1P or its analogs can be used to curtail the invasive and metastatic behavior of cancer cells.
